# Alexander’s Disease: Potential Drug Targets and Future Directions

**DOI:** 10.1007/s12035-025-05083-1

**Published:** 2025-05-31

**Authors:** Emily Zavala, Tahl Zimmerman

**Affiliations:** Biomedical Sciences Program, Department of Physician Assistant Studies, High Point University, High Point, NC USA

**Keywords:** Alexander’s, Neurodegenerative, Treatment, GFAP, Astrocytes, AxD

## Abstract

Alexander’s disease is a rare neurodegenerative disorder primarily characterized by upregulation of the GFAP gene and the formation of Rosenthal fibers. Its prognosis is fatal, with limited treatment options currently available. The GFAP protein is a marker for mature astrocytes. It results in the upregulation of reactive astroglioses. Reactive astroglioses is a neuroprotective condition that, when functioning correctly, helps protect the brain from stress and injury and prevents further injury. However, unregulated reactive astroglioses is linked with many neurodegenerative diseases. Due to the relative rarity in the incidence of AxD, treatment options have not been as widely investigated. This review explores potential drug targets that may impact GFAP gene expression, such as STAT3, GDNF, NF-kB, LCN-2, and the LPS pathway. These drug targets have previously been or are currently being explored in other neurodegenerative diseases such as Parkinson’s disease and Alzheimer’s disease. The only treatment option currently in clinical trial phases involves methods to induce the knockout of the GFAP gene. Due to GFAP’s neuroprotective role in brain injury and stress, it is important to explore alternative treatment options that downregulate GFAP as opposed to shutting it off entirely.

## Introduction

**Fig. 1:**
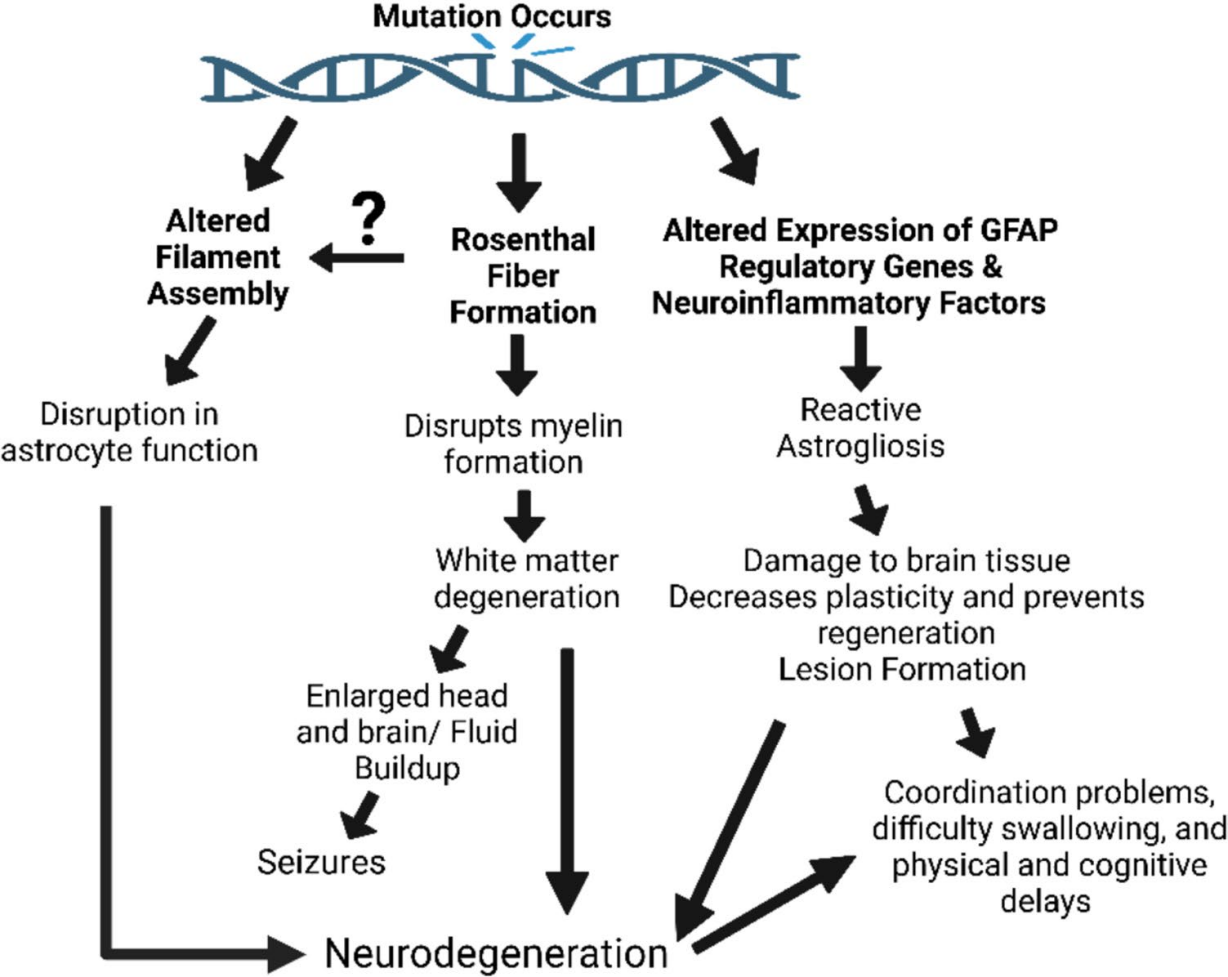
A summary of AxD pathology; connection between observed impact and their corresponding clinical presentation. A mutation occurs in variable locations on the GFAP protein, resulting in the following direct and observed results: altered intermediate filament assembly, Rosenthal fiber formation, and upregulation of GFAP and its respective regulatory genes. As a result, astrocyte function and myelin formation are disturbed, and reactive astrogliosis is upregulated. This results in white matter degeneration and brain tissue damage. These account for the symptoms observed in individuals with AxD, such as coordination problems and developmental delays. All these  outcomes compound to further contribute to neurodegeneration, which in turn further exacerbates these issues

Alexander disease (AxD) is a neurological disorder mainly caused by unique, somatic mutations in the glial fibrillary acidic protein (GFAP) [1]. Rarely inherited, deleterious mutations, or mutations that reduce reproductive fitness and result in premature death, also cause AxD [1]. This condition is marked by the upregulation of the GFAP gene, which has many structural and physiological impacts, such as disruption of the filament assembly network in astrocytes and Rosenthal fiber formation in astrocytes [2]. Although precise mutations in the GFAP gene are variable, all known cases present with an upregulation of the GFAP protein itself. The pathogenesis of non-heritable AxD is unknown, particularly in the case of infantile AxD. However, some studies suggest that environmental factors may contribute to the disorder in adults, such as brain trauma/injury, alcohol exposure, and infection [1]. In adult-onset AxD, current evidence suggests head trauma and injury may be the leading cause of disease onset [3]. Potentially, infantile and juvenile onset believed to be caused by somatic mutations may be caused by heritable mutations in not yet symptomatic parental genomes, as there has been an observed latency period between trauma and disease onset of up to 10 years in adult cases through epigenetic gene alterations [3]. Adult-onset cases may also have mild symptoms that result in a missed or incorrect diagnosis [3, 4]. AxD is separated into two distinct subtypes based on the onset of the disease: type 1 and type 2. Forebrain lesions and early, infantile onset are typical of type 1 AxD, whereas type 2 is characterized by hindbrain lesions with a variable age of onset [2]. Symptoms are variable and depend on age of onset and individual manifestations, but may include macrocephaly, failure to reach milestones, abnormal motor function, and and speech difficulties [5]. The age of onset and severity of symptoms are believed to be factors in the progression speed of the disease. Still, what other factors may play a role is unclear, as severity and age alone are insufficient in determining the rate of disease progression [5]. AxD is commonly diagnosed with magnetic resonance imaging (MRI), which shows abnormalities in brain white matter [5]. Treatment methods have been tested on mouse models, but no standard treatment is available for humans. Recently, GFAP knockout has been explored as an option for treating AxD, a method that is based on the use of antisense oligonucleotides (ASOs) [2]. GFAP knockouts have been shown to prevent disease in rat models and reverse existing pathologies associated with AxD, such as improving myelin degradation [2]. However, this strategy has not reached beyond clinical trials. Although it is a rare disease, it is vital to find new and more efficient treatment methods for those inflicted with AxD. Finding an effective target for treatment may also have further clinical implications. This is because numerous other neurodegenerative disorders show the hallmark upregulation of the GFAP gene observed in AxD, such as Parkinson’s and Alzheimer's, as well as in some cases of non-specific dementia [6]. Finding a treatment for AxD that potentially lowers GFAP expression to normal levels may prove helpful in treating other diseases and reducing their respective symptoms. The purpose of this review is to explore potential drug target options. A summary of the causes of the AxD pathology is provided below in Fig. 1.

### Prevalence, Symptoms, and Prognosis

The exact prevalence of Alexander's disease is unknown, most likely due to its relative rarity. One study suggested a prevalence of 1 in 2.7 million in Japan, but global prevalence has not been determined accurately [5]. Symptoms vary with the age of onset and severity of GFAP overexpression, but some physiological markers are common. AxD is commonly separated into subtypes based on either onset age alone or based on both physiological symptoms and onset age [5]. Other classifications do exist based on neural malformities and affected areas, but these are not generally used in clinical settings [4]. Infantile onset is marked by an appearance from birth to age 2. Juvenile onset is marked by an appearance from ages 2 to 12. Meanwhile, adult onset is marked by onset after age 12 [7]. Infantile onset is characterized by macrocephaly, psychomotor delay, seizures, and spasticity [8]. The basal ganglia and periventricular rim often present with lesions [4]. Juvenile onset is marked by speech problems, difficulty swallowing, poor coordination, weakened muscle movement, seizures, and developmental regression [5]. In the juvenile type, brain stem lesions are often observed and may be mistaken for tumors [4]. Likewise, lesions are often observed in the dorsal medulla [4]. In the adult type, onset and symptoms are variable but lack macrocephaly and are frequently associated with a waddling gait [5]. Adults with AxD may have different degrees and consistency of neurological symptoms [7]. In the adult type, abnormalities have been observed in the cerebellar white matter, sometimes associated with atrophy of the medulla oblongata and cervical spinal cord [4]. Based on onset and physiological symptoms, AxD is categorized into type 1 and type 2 versions of the disease [5]. Type 1 is associated with early onset, seizures, macrocephaly, developmental delay, and failure to reach milestones [5]. Type 2 is associated with later onset, abnormal eye movement, autonomic dysfunction, and uncontrolled movement of the palate muscles, hyperreflexia, and medulla oblongata [5].

Type 1 AxD is associated with forebrain lesions, while type 2 is associated with hindbrain lesions [2], and their respective symptoms may result from this. The forebrain plays a significant role in information processing related to complex cognitive functions such as memory and language, planning and execution of motor activities, regulation of wakefulness, and behavioral responses to emotions [9]. Lesions to areas within the forebrain could account for the developmental delays and for the often severe cognitive issues observed in AxD type 1 patients.

The hindbrain directs functions essential for survival, such as motor activity, respiration, sleep, and blood circulation. It receives and transmits sensory inputs from the auditory and vestibular systems, including respiratory rhythm and motor activity [10]. Lesions to areas within the hindbrain could account for the uncontrolled muscle movements and autonomic dysfunction seen in type 2 AxD.

Infantile, juvenile, and adult-onset cases all present the characteristics observed in all AxD cases: Rosenthal fiber formation, reactive astrocytes, and altered intermediate filament assembly [1].

Alexander's disease is almost always fatal [11]. There is no known cure. Symptoms get progressively worse until fatality [11]. However, progression, speed, and intensity vary widely between individuals and may involve some genetic components [2].

### Diagnosis and Pathogenesis

MRIs are the most used tool to diagnose AxD [1]. The disease presents with white matter degradation in affected areas, such as the hindbrain or forebrain, which can be observed on an MRI [1]. Although other neurodegenerative disorders also present with white matter degradation, therefore, clinicians determine a diagnosis based on the patient's symptoms and the lack of other biomarkers/symptoms associated with other disorders [12]. These unclear diagnostic standards introduce the potential for misdiagnosis and may contribute to the limited understanding of AxD’s pathogenesis. Vague diagnostic standards are not uncommon in neurodegenerative disorders that manifest in infants and juveniles [13]. Due to a combination of their relative rarity in the population, lack of skilled experts on specific disease types, lack of clarity between observed pathology and associated symptoms, as well as unclear pathogenesis, neurodegenerative disorders manifesting in young children do not have a well-defined approach for diagnosis or treatment [13]. These limitations could also contribute to the limited understanding of AxD’s pathogenesis. Although AxD is generally believed to be caused by de novo mutations with only a rare genetic inheritance, studies suggest this assumption may be overly generous [4]. AxD with a later age of onset may be misdiagnosed or have symptoms so mild that the afflicted never seek diagnosis [4]. Rosenthal fiber formation has been observed during autopsy with no prior diagnosis of AxD, suggesting that parents may pass down the disorder genetically without realizing it [4]. In adults, studies have found a linkage between brain injury/trauma and the onset of AxD, with a latency period of up to 10 years [1]. A latency period may mean parents can pass down a GFAP mutation without realizing they are carriers. Gene testing may be employed as a more exact method of diagnosing AxD. Still, its use generally only extends to the afflicted patient, so a heritable mutation may not be identified [4]. Likewise, other disorders show altered GFAP expression, so observing this alone may not be sufficient to diagnose AxD properly [4]. Juvenile neurodegenerative disorders do not typically have a standard approach for diagnosis or treatment, and one study suggests that global standardization of both diagnosis and treatment may be required [13]. A standardization specifically for leukoencephalopathies was proposed, including conducting a full molecular gene analysis [13]. To expand on that proposal, it may prove helpful to do gene testing on parents of AxD patients to rule out that these mutations are not inherited [4]. However, the adoption of genetic screening for rare diseases such as AxD can only work with adequate funding in order to make this tool accessible to patients [14].

### GFAP Protein

Almost all cases of Alexander's disease show a mutation in GFAP that results in overexpression of the GFAP gene. GFAP is a type III intermediate filament that is a marker of mature astrocytes and aids in astrocyte differentiation and reactivity [15]. Abnormally high expression of GFAP protein is consistent with many known neuropathies such as Alzheimer’s disease, Parkinson’s disease, and certain forms of dementia [6]. GFAP is the significant component of the cytoskeleton [15]. It provides structure and support for cells and impacts cell movement, division, and signaling [15]. Due to its high plasticity, GFAP can efficiently alter its polymerization state to account for environmental changes. It also acts as a guiding protein for other proteins through its localization functions [15]. GFAP is also thought to be the primary basis for forming astrocytes, as it forms a significant part of the astrocyte scaffolding network that allows astrocytes to be structurally correct and function correctly [15]. Astrocytes play a crucial role in neuronal function and maintenance. They are involved in a wide range of other neurological processes that affect the formation and elimination of synapses, ionic homeostasis, neurotransmitter clearance, and regulation of the volume of extracellular space [16]. Although unspecified, some studies have suggested that astrocytes may play a role in cognitive function, but whether this role stems from the paticipation of astrocytes in the maintenance of  neuronal health, or via another property of this cell type,  is unclear [16]. The potential role of astrocytes in cognitive function is significant for AxD because many AxD patients present with cognitive delays and/or regression. The GFAP protein is essential for the maintenance of astrocytic structure and shape. It allows astrocytes to form physical barriers between adjacent neurons and stabilize the environment [15]. GFAP also functions as a transport protein guide for astrocytes [15]. GFAP is believed to localize some proteins responsible for the adaptable retraction and extension of the cytoskeletal network, processes that coincide with GFAP reassembling, transporting, and membrane protein recycling machinery [15].

### Common Mutations in GFAP That Lead to AxD

Most cases of AxD are caused by missense, single-nucleotide mutations in the coding sequence, causing a gain-of-function mutation [2]. However, these mutations are not uniform in all cases [2]. Studies suggest that most mutations consistent with AxD result from variable amino acid residual changes to a more nucleophilic amino acid, specifically a histidine or cysteine [17]. Due to its highly reactive thiol group, cysteine residues are more sensitive to oxidative species [18]. Histidine residues are also sensitive to oxidation due to the aromatic side chain they contain [19]. This more nucleophilic amino acid alteration pattern is true for the two most common GFAP mutations, C239R and R239H. The C239R mutation consists of an arginine replaced by a cysteine [17]. The C239R mutation accounts for approximately 20.3% of all cases [20]. The R239H mutation consists of an arginine replaced by a histidine [20]. The R239H mutation accounts for approximately 16.6% of cases [20]. These two most common mutations are the only ones consistently correlated with disease severity and poor prognosis [20].

The oxidation of cysteine residues seen in the most common AxD mutation is also linked to other neurodegenerative diseases as well, such as Alzheimer’s and ALS [18]. This oxidation is believed to cause protein aggregation, which is observed in AxD in the form of Rosenthal fiber formation, and protein misfolding, which could cause the improper regulation of the GFAP gene observed in AxD [18]. One study found that this increased sensitivity of cysteine to oxidation in the C239R mutant contributes to mitochondrial alterations that impact the filament assembly network and increases oxidative stress [17]. This is significant because one of the main hallmarks of AxD is altered filament network assembly, which may account for the astrocyte malfunction seen in AxD patients. When GFAP is overexpressed, the cytoskeleton has been observed to break down into intermediate filament fragments, which results in malformation of the cytoskeleton network [17]. The cytoskeletal network is a vital part of the structure and support of astrocytes, therefore, this alteration of the cytoskeleton network may result in astrocytic malformation. Likewise, because the cytoskeleton network aids cell signaling, its malformation may prevent essential astrocyte functional proteins from behaving correctly.

### Astrocyte Malfunction

In humans, GFAP is believed to be required for reactive astrogliosis, specifically in the extension and thickening of astrocytic processes observed in reactive astroglioses [21]. Reactive astrogliosis is characterized by the proliferation of astrocytes and an increase in the size and activity of astrocytes [21]. Along with altered morphology, reactive astrogliosis is also associated with altered functionality, such as in gene expression [22]. Reactive astrogliosis is a normal stress response to brain injury, and shutting down this process may prove disastrous if trauma were to occur [23]. When appropriately regulated, reactive astrocytes induce alterations in gene expression and cell hypertrophy that limit tissue damage and preserve cellular domains during neural stress [22]. However, improper regulation and persistence of reactive astroglioses is linked to many known pathologies that involve neuroinflammation and neurodegeneration [23]. Mouse models of AxD that contain a GFAP knockout have proven successful at slowing down the progression of the disease and, in some cases, reversing some damage that has already been done, with only a mild impact on injury susceptibility—the ability of the brain to recover from neural stress and injury [2]. However, these methods had previously been widely debated as feasible for humans. This is mainly due to the the elevated complexity of astrocytes in humans compared to mice. Human astrocytes have a 2.55-fold bigger diameter and a 16.5-fold higher cellular volume than rodent astrocytes [24]. Likewise, while human and mouse astrocytes share some common gene expression patterns, there are also thousands of genes with differing expression levels in human astrocytes compared to mouse astrocytes [25]. Perhaps most significantly, human astrocytes show a higher expression of genes involved in the neural stress response, which includes reactive astroglioses [25]. It is important to note that GFAP knockout mouse models have little impact on injury susceptibility. The elevated expression of defense response genes in humans compared to mouse astrocytes suggests that the lack of significant differences in injury susceptibility observed in GFAP knockout mouse models may not accurately reflect the situation in humans. GFAP knockout in humans may negatively affect injury susceptibility more dramatically in humans than in mouse models due to increased defensive gene expression.

### GFAP Protein Structure

GFAP consists of a coiled-coil dimer [26], which may account for its sensitivity to multiple different point mutations that all function to overexpress GFAP in AxD. Coiled-coils are composed of alpha helices that have highly specific interactions and affinities, and are generally well-conserved sequence-to-structure relationships [27]. Because of this virtual invariancy, even a single point mutation that changes a single amino acid may disrupt essential helix-to-helix interactions [27]. This inflexibility could explain why a single point mutation in GFAP can lead to dramatic impacts on the GFAP protein and the subsequent dysregulation of normal GFAP function. Mutations in the GFAP protein that account for the AxD pathology are hypothesized to destabilize the coiled-coil, which results in the loss of stabilizing interactions in the dimer and tetramer, accounting for the altered filament assembly seen in AxD patients [26]. One possible explanation for this malformed cytoskeletal network may be GFAP’s role in localizing proteins essential to maintaining astrocyte structure. As a result of an altered amino acid sequence, new protein–protein interactions between GFAP and other proteins could occur, while normal interactions could be disrupted. Altered protein–protein interactions could result in improper signals to vital proteins necessary for possible filament formation. It is unclear why these destabilizations result in an increased concentration of GFAP.

### From Mutation to Disease

Although not all pathologies are present in every case, most AxD mutations have standard maladaptive features. These include altered cytoskeletal filament assembly, Rosenthal fiber formation [2], and altered expression of GFAP regulatory genes and neuroinflammatory factors [28].

Rosenthal fibers are inclusions found in the cytoplasm of astrocytes, and their formation is pathognomonic to AxD [29]. Rosenthal fiber composition is variable and generally composed of multiple types of protein, such as cytoskeleton scaffold proteins, heat shock proteins, ubiquitin, and intermediate filaments like GFAP [2]. Little is known about how these fibers appear in disease states, but high levels of these fibers are generally not found in healthy tissue. Since Rosenthal fiber formation is often associated with the areas of the brain that are high in white matter, some have suggested the formation of these fibers is linked to the white matter degradation observed in AxD [30]. However, Rosenthal fibers and white matter degradation do not always coincide in AxD pathologies, even in areas of high Rosenthal fiber formation [30]. One possibility is that the malformation of the cytoskeleton intermediate filaments caused by the upregulation of GFAP formation accounts for the Rosenthal fiber formation observed in AxD, as intermediate filaments are one of the many components found in Rosenthal fibers.

Like Rosenthal fiber formation, the white matter degradation and myelin malfunction associated with AxD are of unknown mechanistic origin [31]. One study found that rat models of AxD displayed significant decreases in white matter volume, myelin proteins, and thinner myelin sheaths and an increase in the degradation of axons in the spinal cord [31]. Myelination is crucial for the proper connections through the Central Nervous System (CNS) [32]. Disruption of this system in AxD may account for most patients' delayed cognitive abilities and cognitive regression. Astrocytes also play a significant role in maintaining white matter volume through regulating ion and neurotransmitter balance and secretion of myelin-promoting and inhibiting factors [31]. The widespread role that astrocytes have played in the neural system makes it difficult to determine if the white matter degradation directly results from astrocyte malformation due to GFAP dysregulation or if white matter degradation results from the appearance of Rosenthal fibers in white matter–rich areas. Both processes may contribute to the degradation seen in AxD.

Altered expression of GFAP regulatory genes, such as STAT3, and other neuroinflammatory factors are also observed in the AxD pathology [33]. Still, the exact mechanism responsible for changes in expression patterns is unclear [33]. Studies are inconclusive regarding whether overexpression of GFAP regulatory genes leads to overexpression of GFAP or vice versa [33].

### Physiological Impacts of GFAP Overexpression

As noted, GFAP overexpression is thought to contribute to the malformation of the cytoskeleton network in astrocytes. However, another impact of GFAP overexpression is a steep increase in reactive astroglioses. Reactive astrogliosis is a stress response invoked by neurological trauma, which, when appropriately regulated, helps to limit tissue damage and restore homeostasis [23]. However, the persistence of over-reactivity of astrocytes proves harmful to brain tissue and function due to decreased neural plasticity and the inhibition of regeneration responses [23]. It is interesting to note that although the upregulation of GFAP in healthy individuals who experience brain injury is accompanied by an upregulation of brain-derived neurotrophic factor (BDNF), in other neurodegenerative disorders, increased GFAP expression has been observed to be accompanied by a decrease in BDNF [34]. BDNF is known for its role in neural plasticity, and a reduction in BDNF activity may account for the symptoms seen in infantile and juvenile AxD, as neural plasticity and activation are vital at that age for proper development [35]. One study that focused on epilepsy, a neurological disorder, found that immediately after an epileptic episode, BDNF levels increase but eventually fall back to normal levels, indicating its role in neuroprotection [35]. Reduced BDNF levels are observed in other neurodegenerative diseases such as Alzheimer’s. This reduced BDNF pattern may also be true for AxD, which could contribute to the pathologies observed in the disease condition [35].

Although Rosenthal fiber formation consistently correlates with brain malfunction and disease, the exact downstream pathways causing these brain malfunctions are unknown [2]. One speculation is that the buildup of these proteins prevents the normal distribution of the cytoskeletal network. Rosenthal fibers are more resistant to solubilization than other astrocytic components such as GFAP, so this may emphasize the overexpression of GFAP and compound the issue of GFAP overexpression seen in AxD [2]. The further upregulation of GFAP may cause maladaptive traits in neurological disorders that contribute to decreased neural activity and increased neural degeneration.

Mitochondria dysfunction may also exacerbate GFAP overexpression. One study on the R239C mutant showed that mutant cells had longer mitochondria with larger volumes than seen in wild-type cells [33].

### Current Symptomatic Treatments

There is no known cure for AxD and no standard treatment protocol. Nearly all treatments aim to support and ameliorate symptoms associated with the disease, such as physical therapy, removal of excess neural fluid, and anti-seizure medications [1]. The only current treatment option being tested in clinical trials is GFAP knockout ASOs [2].

### Current GFAP Knockout Treatment

A current treatment option still in the early utilization phases is GFAP knockout/inhibition using targeted antisense oligonucleotides [2]. In R237H mutant rat models, knocking out GFAP has been shown to prevent disease altogether [2]. In more advanced rat models that have the R237H mutation, severe motor malfunction associated with a worse fatality prognosis is observed. Meanwhile, in the R237H model, the GFAP knockout was observed to reverse the myelin degradation seen in AxD and the motor impairment [2]. Symptoms in rat models more closely mimicked those seen in human patients compared to mouse models, including deficits in motor function not seen in mouse models [31]. However, it should be noted that, unlike humans, rats did not show an elevation in GFAP found in the spinal cord [31]. It is also essential to note that rats with the AxD pathology could reach typical developmental milestones before failing to thrive, unlike infants who present with AxD [2]. Rats who received the antisense oligonucleotide treatment showed suppressed levels of GFAP, improved motor function, decreased neuroinflammation, normalized astrocyte-related protein levels, and increased white matter [31]. GFAP suppression in rat models also showed no apparent negative impacts on injury susceptibility [31]. This course of treatment has recently moved to clinical trials and is currently the most promising treatment strategy [2, 31]. However, other targets may be required since mouse and rat models do not accurately reflect the development of AxD as seen in humans. Mouse and rat models also may not represent GFAP’s presumed role in reactive astrogliosis well. As noted, reactive astroglioses is a normal stress response to neural injury. Although no overt effects are observed in mice and rat models, the higher complexity of astrocytic networks in humans may prove an obstacle to GFAP suppression. GFAP expression naturally increases with age in humans [36]. The reason for this increase is unclear, so suppressing GFAP in later stages may result in unexpected adverse side effects not observed in rodent models. Although an increase in GFAP expression is also observed in rat models and GFAP knockouts in rats showed little impact on injury susceptibility [31, 36], the prolonged lifespan of humans as compared to rats may prove problematic if increased GFAP expression is linked to a higher requirement of neural protection as we age. Indeed, GFAP knockout mice who do not have the AxD pathology and GFAP overexpression show a greater susceptibility to brain injury [6]. Likewise, neuronal stress positively correlates with increased GFAP expression, indicating a role for GFAP in neuroprotection [6].

### Potential Future Targets for Treatment Development

Due to its relative rarity, limited information is available about AxD. The limited information means that potential treatments still need to be explored. Many molecules contribute to GFAP formation and regulation, and targeting these may prove helpful in alleviating the detrimental symptoms associated with AxD. A list of GFAP modulators and potential targets for AxD treatment were compiled (Table 1). This review focuses on the modulators that have been studied in other neurodegenerative diseases for their therapeutic potential, as well as those that have been studied in models of AxD itself. The LPS pathway has not been explored extensively as a drug target on its own. Still, the possible downregulation of the LPS pathway by altering other GFAP modulators such as LCN-2, STAT3, and NF-kB is discussed.

**Table 1:** A list of current known modulators of GFAP and their respective roles. Bolded modulators are the focus of this review as potential drug targets due to the available information regarding other neurodegenerative diseases or because current research on their role in Alexander's Disease is available. These have been linked to GFAP expression or are upregulated with GFAP upregulation

Modulators	Role	Effect	Source
**STAT3**	**Transcriptional activation**	**Upregulation**	[33]
CNTF	Transcriptional activation	Upregulation	[37]
Glucocorticoids/thyroid hormones	Transcriptional activation	Upregulation	[37]
**GDNF/neurturin**	**Transcriptional activation/regulatory**	**Promote GFAP transcription and stabilize GFAP levels**	[21]
AP1	Faciliatory for gene transcription	Aids in switching on GFAP upregulation	[15]
C-Jun	Faciliatory for gene transcription	Aids in switching on GFAP upregulation	[15]
PPAR-y	Faciliatory for gene transcription	Both upregulates and downregulates GFAP expression	[15]
**NF-kB**	**Faciliatory for gene transcription**	**Transcriptional activation → activates the LPS system**	[15]
BRG1	Faciliatory for gene transcription	Promotes GFAP aggregation and expression	[15]
**Lipocalin (LCN-2)**	**Faciliatory for gene transcription**	**↑ Lipocalin**	[38]
		**↑ GFAP expression**	
**LPS**	**Transcriptional activation**	**↑ Neuroinflammation**	[21]
		**↑ TRPV1 (alleviates neuroinflammation)**	
Synemin, vimentin, nestin	Intermediate filaments	Upregulated with GFAP	[39]
Smad1	Faciliatory for gene transcription	Stimulates GFAP expression with BMP2 interaction	[40]
BDNF	Aids in brain plasticity and neuronal excitement	Upregulated by GFAP activity; downregulated with abnormal GFAP expression	[34, 35]

### STAT3 Transcription Factor

STAT3 is a known cytoplasmic transcription factor that modulates GFAP formation [33]. Some studies found that STAT3 directs the increase and accumulation of GFAP observed in AxD patients [33]. During the inflammatory response, STAT3 is phosphorylated and translocates into the nucleus, where it promotes the expression of genes that secrete cytokines and control other pro-inflammatory processes [41]. STAT3’s role in inflammation is achieved through the JAK2/STAT3 pathway. STAT3 is recruited to a phosphorylated JAK2 dimer and phosphorylates itself on a tyrosine residue [41]. Once phosphorylated, homodimerization or heterodimerization occurs via attachment to the SH2 region of one monomer or other STAT3 molecules. The activated STAT3 molecule translocates to the nucleus and attaches to DNA regulatory genes, some of which stimulate the expression of cytokines [41]. GFAP is one of these regulatory genes that STAT3 activates [33]. The upregulation of STAT3 has been linked to other neurodegenerative disorders like Alzheimer's and Huntington’s disease and has been suggested as a possible drug target in the former [41]. In a healthy individual, STAT3 regulates GFAP levels during astrocyte development by binding to its promoter region and inducing expression [33]. Some studies have found a linkage between STAT3 and reactive astroglioses, as it has been shown to drive the activation state of microglia in several injury models and to contribute to neurodegeneration [33].

STAT3 has been found to have a twofold increase in expression in both human and mouse models of AxD. The active phosphorylated form of STAT3 (pSTAT3) increases 15- to 40-fold in these disease models. Although STAT3 directly controls GFAP transcription, it is unclear how much is necessary for GFAP production, as GFAP is still overexpressed even when STAT3 is present in only very low levels [33]. Likewise, although GFAP expression is dramatically reduced when STAT3 is completely knocked out, low levels can still be observed. Other transcription factors are also overexpressed alongside STAT3 and GFAP, and because STAT3 is a pleiotropic factor, it may contribute indirectly to GFAP formation [33]. If STAT3 were to activate some other transcription factor that is the genuine activator of GFAP production, it would still be consistent with low levels of GFAP when STAT3 is knocked out. This is consistent with the fact that GFAP is overexpressed even when only low levels of STAT3 are present. Therefore, the extent to which STAT3 is required for GFAP production has not yet been determined [33].

Despite GFAP overexpression still being present in conditions with only low levels of STAT3, because of STAT3’s role in GFAP transcriptional activation and because it has been shown to drive reactive astroglioses, knocking out STAT3 is a possible treatment technique that warrants further investigation. Complete knockout of STAT3 decreased GFAP concentration in tissue models [33]. However, because STAT3 is a pleiotropic transcription factor, complete knockout is lethal in mouse models [33]. STAT3 inhibition has also been explored, such as knocking out only one allele. STAT3 inhibition is a common cancer treatment and has proven successful in inhibiting cell growth. However, mouse models show that even when STAT3 is only expressed in low levels, GFAP is still overexpressed, leading to the symptoms observed in AxD [33]. Reducing levels of STAT3 to a concentration in which GFAP overexpression is depleted is a lethal strategy [33]. Therefore, it is not easy to find a way to maintain STAT3 expression to achieve therapeutic levels of GFAP.

Although STAT3 inhibition has proven to be a limiting factor in AxD regarding GFAP formation, it does not mean it is not still a viable treatment path. Inhibiting STAT3 in mouse models of Alzheimer’s disease leads to decreased pro-inflammatory cytokines and marked decreases in the LPS signaling pathway [42]. The LPS pathway induces microglial activation [42]. Likewise, inhibiting the JAK3/STAT3 pathway in Huntington’s disease resulted in decreased reactive astrocytes, which led to a decrease in Huntington aggregates, a hallmark of that disease [41]. Inhibition of STAT3 results in the same overall effect in both pathologies: a reduction of reactive astrocytes. This inhibition results in a decrease of Aβ plaques and Huntington aggregates in Alzheimer’s and Huntington’s disease, respectively. These outcomes indicate that targeting the STAT3 pathway may prove beneficial in different ways for specific neurodegenerative disorders, including AxD. Consequently, inhibiting STAT3 in AxD may alleviate reactive astrocytes, as observed in Alzheimer’s and Huntington’s, but may also potentially decrease the formation of Rosenthal fibers in AxD. Furthermore, even if STAT3 does not necessarily lower GFAP production to normal levels, as suggested in previous studies, it may still have the potential to limit the neuroinflammation that GFAP contributes to. This reduced neuroinflammation was observed in one study using AxD mouse models, which found that a conditional knockout of STAT3 prevents the reactive astroglioses response alongside some decrease in GFAP expression [33]. A reduced reactive astroglioses response via STAT3 knockout may help alleviate the symptoms of AxD associated with that inflammation, even if the overall GFAP expression is not significantly lower.

### Lipocalin

Lipocalin-2 (LCN-2) is a circulatory protein commonly used as a biomarker for neurodegenerative diseases characterized by inflammation. LCN-2 is a good biomarker due to its role in the inflammatory response [38]. LCN-2 acts as a mediator in reactive astrocytes. Increased levels of LCN-2 are observed in astrocytes exposed to inflammatory stimuli [38]. This increase in LCN-2 during inflammation is significant in AxD mainly due to the reactive astroglioses observed in its pathology. It is believed that the reactive astroglioses contributes in some way to the cognitive breakdown observed in AxD. Reactive astrocytes are generally believed to come in two forms, A1 and A2, with A1 being associated with neurotoxicity and A2 associated with neuroprotection [43]. Although LCN-2 has implications for both neuroprotection and neurotoxicity, studies have found that LCN-2 levels are increased in A1 cells compared to A2 cells [43]. An LCN-2 increase in A1 cells, which is associated with neurotoxicity, suggests that LCN-2 strongly induces the formation of A1 astrocytes over A2 astrocytes. One potential way to treat AxD may be to convert A1 astrocytes triggered by LCN-2 into A2 cells associated with neuroprotection [43]. One possible way to facilitate this conversion from A1 cells to A2 cells is by inhibiting pro-inflammatory cytokines that trigger A1 conversion, which would cause A1 pro-inflammatory cells to revert to A2 anti-inflammatory cells [43]. Interferon regulatory factor 3 (IRF3) has successfully inhibited the gene expression of these pro-inflammatory cytokines and, therefore, aids in converting A1 to A2 cells [43]. Conversion of A1 cells into A2 cells in AxD patients may reduce the neurotoxic effects of A1 cells and increase the neuroprotective effects of A2 cells. One hallmark of AxD is white matter degradation, which contributes to cognitive dysfunction [2]. Increased LCN-2 levels are associated with white matter degradation in optic nerves compared to tissues with LCN-2 knockout models [44]. Likewise, LCN-2 levels were increased in AxD tissue samples and may contribute to the white matter degradation observed [44]. Although not attempted in all cell types, an LCN-2 knockout has been attempted in an experimental autoimmune optic neuritis (EAON) model. Even though this disease condition does not share many characteristics of AxD, they do share the following distinct characteristics: myelin degradation, reactive astroglioses, overexpression of inflammatory factors, and destructive inflammatory conditions [2, 23, 44]. When LCN-2 was knocked out in the EAON model, all the shared characteristics mentioned above were decreased. It was also theorized that LCN-2 played a significant role in the initial onset of EAON, which may also be true in AxD [44]. Suppressing LCN-2 may prove helpful in alleviating these symptoms in AxD as well as its associated cognitive decline. It may also help prevent progression of the disease in late-stage onset cases if, as hypothesized, it plays a significant role in the onset of the disease. The theory that LCN-2 suppression can slow down disease onset and cognitive decline is further supported by the fact that LCN-2 levels are at their highest in the early stages of cognitive decline in Alzheimer's disease [43]. Increased LCN-2 levels are associated with reactive astroglioses since activated astrocytes secrete LCN-2 [43]. Although EAON and Alzheimer’s disease do not share all the same hallmarks as AxD, it is worth noting that both diseases show an upregulation in reactive astroglioses similar to that seen in AxD and targeting LCN-2 levels in the previously mentioned studies were all aimed at modulating this reactive response. Therefore, even with the differing characteristics of each disease, it is possible that targeting a similar problem—in this case, reactive astrogliosis—may still prove helpful in eliminating the symptoms and severity of AxD.

### GDNF Protein

Similarly to LCN-2, GDNF has been shown to play a pivotal role in regulating neuroinflammation in many neurodegenerative diseases such as Parkinson’s, Alzheimer’s, and ALS [45]. It is one of the major secretory substances released by glial cells, including astrocytes, and has been associated with neuroprotection in multiple disorders [38]. GDNF’s expression in AxD has not been extensively studied, but its role as a modulator of GFAP is relevant. GDNF has a role in increasing and decreasing transcription of GFAP, depending on the cellular environment in the neuronal membrane where GDNF is located [21]. Although GDNF has been shown to promote GFAP transcription in injury models, thus increasing GFAP levels, GDNF has also been known to regulate GFAP levels back to pre-injury levels [21]. GDNF has been appreciated for its neuroinflammatory protective role and has been suggested as a potential drug before, but never has this idea been applied specifically to AxD. Neuroinflammation is a hallmark of AxD and most likely contributes to nearly every physiological impact observed in the pathology. Neuroinflammation also plays a role in regulating GFAP levels to pre-injury levels. Therefore, we suggest that modulating this protein may be a therapeutic approach in AxD as well.

Studies in Parkinson's models have shown that GDNF inhibits microglial activation [36] and decreases the characteristic degradation of dopaminergic neurons [45]. Dopaminergic neurons are found primarily in the midbrain region, while AxD lesions are believed to be limited to the hindbrain and forebrain regions. However, since microglial activation is inhibited in Parkinson's models, GDNF may still help alleviate the neuroinflammation observed in AxD.

It is worth noting that human-based clinical trials regarding GDNF infusion and gene delivery into the CNS for Parkinson's patients have provided conflicting and disappointing results when compared to pre-clinical trial data [46]. Although it is unclear why, some patients developed GDNF antibodies following infusion [46]. This antibody formation may be because the influx of GDNF signals to the immune system that something is wrong, and antibodies are created to combat this response. Reaching the target region also proved somewhat of an obstacle. GDNF does not bypass the blood–brain barrier [46]. Although patients did report an increase in motor function, the results of all clinical trials failed to reach their goals [46]. However, GDNF therapy is still considered a potential therapeutic approach because of its role as a neuroprotectant. Therefore, it may be worthwhile to assess the therapeutic potential of GDNF in AxD as well. Parkinson’s studies focus mainly on dopaminergic neuron degradation and regeneration. Although dopaminergic neuron degradation is not a hallmark of AxD, the role of GDNF in GFAP expression is still noted. It may prove more straightforward to reach target tissues in the case of AxD. Moreover, the results may be more promising since the exact mechanism and role of GDNF in neurodegenerative disorders is not known. Although mainly found in the striatum in the midbrain region, where the dopaminergic receptors targeted in Parkinson’s are found, GDNF is also expressed in the thalamus and septum. The thalamus and septum are both found in the forebrain [47]. Since GDNF is expressed in forebrain regions, this is another strong indication that GDNF treatment in AxD patients may prove helpful in alleviating symptoms. Furthermore, GDNF treatment may help reverse the effect of the disorder, as type 1 AxD is characterized by forebrain lesions. Targeting the forebrain with GDNF treatment is especially relevant because type 1 is generally considered to have a worse prognosis [5]. Individuals who have infantile-onset type 1 AxD succumb to the disease within weeks to a few years [5]. A twofold mortality rate is observed in type 1 as compared to type 2 [5].

### Nuclear Factor Kappa B

The NF-kB pathway is a diverse and complex signaling pathway associated with several aspects of the immune response [48]. The NF-kB pathway induces the transcription of inflammatory cytokines and activates innate immune and T cells [48]. When functioning normally and appropriately regulated, the NF-kB pathway is a protective response to fight off infection and heal damaged tissue [48]. However, uncontrolled NF-kB activation is associated with many pathologies, including neurodegenerative diseases [48]. Although NF-kB levels have not been explicitly studied in AxD, the GFAP promotor region contains NF-kB binding sites [49]. Increased astrocytic NF-kB levels positively correlate to increased GFAP levels [50]. It is also noteworthy that recent studies have found a linkage between NF-kB and the learning/memory processes [51]. Increased NF-kB is associated with increased GFAP levels and learning/memory, which is important because cognitive dysfunction and developmental delay are both symptoms commonly associated with AxD. Although there is no current information regarding whether NF-kB is dysregulated in AxD, there is the possibility that dysregulated NF-kB may interfere with infant development by being unable to contribute to the learning/memory processes required. If NF-kB cannot appropriately regulate learning and memory, it may result in or exacerbate the symptoms seen in AxD. NF-kB is typically inactive in the cytoplasm [52]. To be activated, NF-kB relies on inhibiting its inhibitory proteins, IκBs [52]. The inhibition of IκBs is done by activating the IKK2 complex [52]. The IKK2 complex phosphorylates IkBs and targets them for destruction. When IkBs are destroyed, they cannot inhibit NF-kB activation [52]. The multiple steps required for NF-kB activation essentially gives three therapeutic potentials in decreasing NF-kB activity and, theoretically, decreasing GFAP production: inhibiting the NF-kB pathway itself, upregulating IκBs to keep NF-kB in its inactive form, or inhibiting IKK2 so it, in turn, cannot inhibit the IkBs that inhibit NF-kB. Inhibiting NF-kB is critical due to the pathway's differing role in neurodegenerative disorders.

### NF-kB in Alzheimer's and Parkinson’s Disease

Inhibiting NF-kB to treat neurodegenerative Alzheimer’s disease has been previously explored as a treatment option due to its role in promoting the activation of multiple molecules that behave erroneously in this disease condition [50]. Although not all molecules downstream of NF-kB are relevant to AxD, the inflammatory effect potentially caused by or exacerbated by NF-kB is seen in both pathologies and is believed to contribute to the cognitive loss observed in both. In an Alzheimer's disease model, mice exposed to NF-kB inhibitory molecules were seen to have a decrease in pro-inflammatory processes, synaptic toxicity, and cognitive decline [50]. However, some studies suggest that NF-kB plays a role in clearing the Aβ plaques that are a hallmark of Alzheimer’s [50]. Long-term inhibition of NF-kB may prevent plaque clearance, with negative consequences for the healing process [50]. Although Aβ plaques are not observed in AxD, it is worth noting that inhibiting NF-kB may have detrimental effects on systemic health. One option for preventing these negative effects is to promote the inhibition of only astrocytic NF-kB. If only astrocytic NF-kB is inhibited, it may regulate the reactive astroglioses and improve symptoms of AxD without causing a body-wide downregulation of GFAP.

NF-kB can be regulated indirectly as well. For example, IKK2 is necessary for the inactivation of IkB and, thus, for the activation of NF-kB [53]. IKK2 was tested as a target in a Parkinson’s mouse model, resulting in decreased neuronal loss and apoptotic gene expression [53]. Only IKK2 specific to astrocytes was modulated, preventing detrimental effects on the immune system elsewhere. In mouse models treated with IKK2 inhibitor, the decreases in neuronal loss and apoptotic gene expression are directly correlated to reduced levels of gliosis and inflammatory gene expression [53]. Gliosis and inflammatory gene expression are a property of AxD as well and directly contribute to the symptoms observed [53]. This suggests that IKK2 inhibition could be a therapeutic target for AxD as well.

### The Therapeutic Potential of NF-kB in AxD

As previously stated, NF-kB plays a critical role in the immune response by activating innate immune and T cells. Completely inhibiting this pathway may leave the individual vulnerable to disease and infection. NF-kB inhibition also can limit brain tissue healing in unrelated brain injuries. NF-kB plays a role in inducing the transcription of inflammatory cytokines, which, in turn, trigger reactive astroglioses, a protective response when regulated appropriately [48]. One way to prevent this vulnerability to disease is by only directing NF-kB inhibition in astrocytes, thus keeping the immune system functioning normally in other body parts. However, this is not a perfect solution. Limiting NF-kB in astrocytes may limit the healing ability of brain tissue in unrelated brain injuries. A reduced ability to heal neural tissue could be detrimental to overall brain health, even if AxD-related conditions are improved. Further studies are required to see if vulnerabilities to brain injury are pronounced in NF-kB knockout models. One possible solution for this issue may be to selectively suppress NF-kB in astrocytes and affected tissues rather than completely knocking it out. However, further research needs to be conducted. Notably, preventing the activity of NF-kB has been attempted and has been shown to reduce GFAP levels [49]. One study found that aspirin, a common drug used to prevent NF-kB activity, decreased GFAP levels in treated cells [49]. This decrease in GFAP levels was a result of NF-kB inhibition [49]. Reduction in GFAP levels is significant because it suggests that the inhibition of NF-kB directly affects GFAP expression. Perhaps most importantly, NF-kB inhibition led to the downregulation of GFAP without completely blocking expression of GFAP [49]. Under these conditions, GFAP might still be able to partially perform its neuroprotective role. Further studies need to be done to determine if this decrease in expression can reverse or improve the symptoms associated with AxD.

### Potential Future Treatments: An Additive Effect

Several potential treatment targets were outlined above, all of which impact GFAP expression and contribute to different aspects of other non-GFAP-related neurodegenerative disorders. However, it is also important to note that many of these targets interact with one another, impacting either the expression of one another or their downstream pathways. Inhibiting a single drug target may prove helpful in inhibiting another potential drug target. An additive effect may improve symptoms or slow progression faster than if only one target were affected.

Activated astrocytes upregulate NF-kB transcription, further promoting the inflammatory response [53]. Targeting NF-kB and inhibiting this reactive effect may also limit the expression of other molecules that NF-kB upregulates, such as STAT3 [54] and LCN-2 [38]. Similarly, targeting STAT3 and LCN-2 may result in NF-kB downregulation by limiting reactive astroglioses. GDNF is a potent inhibitor of reactive astroglioses. Therefore, GDNF treatment may result in the downregulation of STAT3, LCN-2, or NF-kB. This is important for two reasons. One reason is that the combined impact of the downregulation of STAT3, LCN-2, and NF-kB that contribute to neurodegeneration may have a potentially additive effect. This additive effect may give a more pronounced decrease in the pathologies associated with AxD. The second reason is that having multiple interacting pathways offers alternative methods of regulating dysfunctional pathways without outright shutting them off. If important pathways are not shut off entirely, it may prevent the negative impacts of complete inhibition. Avoiding complete shutdown of certain pathways can also prevent the inhibition of unrelated downstream pathways that would be affected.

Another example is that LCN-2 is positively regulated by the STAT3 and NF-kB pathways [38]. Therefore, downregulating any of those may also downregulate LCN-2 without needing additional interference, thus further relieving the symptoms seen in AxD.

Finally, many of the modulators already mentioned play a role in activating the lipopolysaccharide-induced pathway (LPS). LPS induces inflammation by upregulating inflammation-related enzymes in microglia, thus resulting in reactive astrocytes [55]. Inhibition of STAT3 decreases LPS-induced microglial activation and reduces pro-inflammatory cytokine concentration in the brain normally induced by the LPS pathway [42]. Importantly, LCN-2 is also positively regulated by the LPS pathway [31]. Finally, the LPS pathway plays a role in activating the NF-kB pathway [56].

**Fig. 2:**
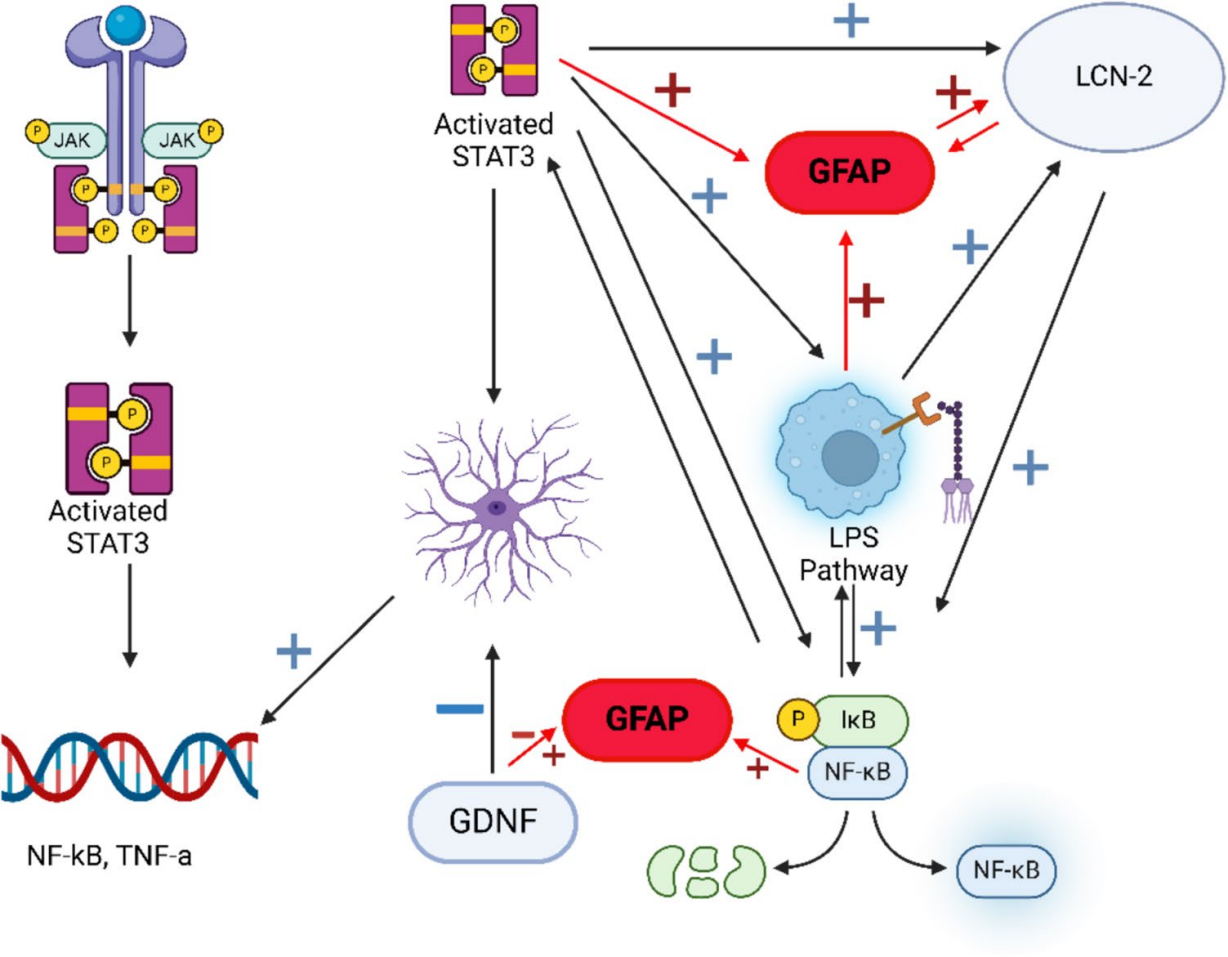
Interactions between the potential drug targets for AxD. Activated STAT3 contributes to reactive astroglioses, LPS pathway, and LCN-2 and upregulates GFAP. NF-kB is upregulated by and upregulates the LPS pathway and STAT3. LCN-2 upregulates NF-kB and upregulates GFAP itself. Lipocalin, LCN-2, is upregulated by the LPS pathway and STAT3 and upregulated by and upregulates GFAP itself. GDNF has a neuroprotective role and decreases reactive astroglioses. It can both upregulate and downregulate GFAP, depending on conditions

One problem with pursuing an additive effect of different GFAP modulators is that the downregulation of multiple inflammatory systems and promoters of reactive astroglioses may result in a weakened response to brain injury and susceptibility. Reactive astroglioses is a stress response invoked by neurological trauma that helps to limit tissue damage and restore homeostasis [23]. This healing process invoked by reactive astrocytes is dysfunctional in most neurodegenerative diseases and proven to be dysfunctional in AxD. However, inhibiting this pathway may very well leave the brain vulnerable to injury and reduce the healing properties of brain tissue. This concern regarding neural vulnerability and reduction in healing potential is also applicable to the downregulation of GFAP itself. However, because the evidence indicates that all these factors are upregulated and dysfunctional in AxD, further research is still needed to determine if inhibition could bring upregulated modulators back to normal expression levels. A visual representation of how these pathways all function to regulate GFAP is shown below in Fig. 2.

## Conclusion

AxD is a neurodegenerative disorder whose mechanisms and symptoms are not clearly understood. Perhaps because of the vagueness surrounding its mechanism and symptoms—and its relatively low prevalence—treatment options are limited. Besides symptomatic treatment, all current treatment options focus entirely on downregulating the GFAP gene. However, GFAP’s role in injury may mean inhibition increases vulnerability to brain injury and has a less effective healing response in associated brain tissues. Therefore, exploring other treatment options is vital. GDNF, NF-kB, Lipocalin, and STAT3 are suggested due to their role in neuroinflammation and neurodegeneration, as well as the fact that they have all been previously studied in other neurodegenerative diseases.

## Data Availability

No datasets were generated or analysed during the current study.
